# High Intensity Exercise: Can It Protect You from A Fast Food Diet?

**DOI:** 10.3390/nu9090943

**Published:** 2017-08-26

**Authors:** Christian Duval, Marc-Antoine Rouillier, Rémi Rabasa-Lhoret, Antony D. Karelis

**Affiliations:** 1 Department of Exercise Science, Université du Québec à Montréal, Montreal, QC H3C 3P8, Canada; duval.christian@uqam.ca (C.D.); ma.rouillier@gmail.com (M.-A.R.); 2 Centre de recherche de l’Institut universitaire de gériatrie de Montréal, Montreal, QC H3W 1W5, Canada; 3 Institut de Recherches Cliniques de Montréal (IRCM), Montreal, QC H2W 1R7, Canada; Remi.Rabasa-Lhoret@ircm.qc.ca; 4 Department of Nutrition, University de Montréal, Montreal, QC H3T 1A8, Canada

**Keywords:** body composition, energy expenditure, energy intake, lipid and inflammation profile

## Abstract

The purpose of this study was to assess the ability of high intensity exercise to counteract the deleterious effects of a fast food diet on the cardiometabolic profile of young healthy men. Fifteen men were subjected to an exclusive fast food diet from a popular fast food restaurant chain (three extra value meals/day + optional snack) for 14 consecutive days. Simultaneously, participants were asked to perform each day high intensity interval training (HIIT) (15 × 60 sec sprint intervals (~90% of maximal heart rate)) on a treadmill. Fast food diet and energy expenditure profiles of the participants during the intervention were assessed as well as body composition (DXA), cardiometabolic profile (lipid, hepatic enzymes, glycated hemoglobin, glucose, insulin, hsC-reactive protein (hsCRP) and blood pressure) and estimated maximal oxygen consumption (VO2 max) pre- and post-experiment. We found significant improvements for fat mass, lean body mass, estimated VO_2_ max, fasting glucose, serum lipoprotein(a) and hsCRP after the intervention (*p* < 0.05). HDL-cholesterol significantly decreased (*p* < 0.002), but the triglycerides/HDL-cholesterol ratio did not change. All other cardiometabolic variables measured remained stable, which includes the primary outcome: the HOMA index (pre: 1.83 ± 1.2 vs. post: 1.54 ± 0.7 values; *p* = 0.35). In conclusion, in large part, insulin resistance and the cardiometabolic profile of young healthy individuals seems to be protected by HIIT from a fast food diet.

## 1. Introduction

The consumption of fast food has considerably increased during the last few decades [1,2,3]. There is evidence to suggest that 36% of US adults consume fast foods during any day of the week [2]. In fact, during 2007–2010, US adults consumed ~11% of their total daily energy intake from fast foods [4]. In addition, several studies have shown that frequent consumption of fast foods may be associated with weight gain [5,6,7] and cardiometabolic complications [7,8]. Indeed, Alhéritière et al. [9] reported a strong positive correlation between the number of McDonald’s restaurants and the prevalence of overweight individuals across 44 countries.

In the general population, there is a popular belief that fast food consumption is generally recognized to be unhealthy irrespective of the calories ingested or the levels of physical activity of an individual. This is not a trivial argument. Interestingly, a recent study showed that short term (seven days) overfeeding (50% increase in energy intake) combined with a reduction in physical activity (<4000 step/day) was associated with a decrease in insulin sensitivity in young healthy men. However, when daily continuous running on a treadmill at a high intensity (45 min at 70% VO_2_ max) was added, this was able to offset the deterioration in insulin sensitivity despite an energy surplus [10]. This provides tantalizing evidence of the protective effects of daily high intensity exercise.

High intensity interval training (HIIT) has been reported to be effective, well-tolerated and a more powerful stimulus than traditional moderate intensity exercises in reducing cardiometabolic disturbances (e.g., blood pressure, lipid profile and insulin resistance) and increasing cardiorespiratory fitness levels in different at risk populations with cardiometabolic diseases [11,12,13]. Thus, a critical question that remains unresolved is whether HIIT could protect the cardiometabolic profile against the potential negative effects of a fast food diet. Knowledge on the effect of a fast food diet in combination with HIIT on the cardiometabolic profile may help us better understand the risk of disease of an individual and as such provide useful information to health professionals. Therefore, the purpose of the present study was to investigate the role of HIIT during a fast food diet on cardiometabolic risk factors in young healthy men. We hypothesized that HIIT would protect the cardiometabolic profile against the potential negative effects of a fast food diet. Specifically, we hypothesized that insulin resistance (the HOMA index as primary outcome), fasting glucose and insulin as well as the lipid and inflammation profile (as secondary outcomes) would not deteriorate after the intervention.

## 2. Materials and Methods

**Participants:** Sixteen participants were recruited in this interventional study between June 2015 and March 2016 using advertisement via emails and short presentations in classrooms at the Université du Québec à Montréal. Participants were included in the study if they met the following criteria: (1) male; (2) aged between 18–30 years old; (3) a body mass index (BMI) between 18.5–29.9 kg/m^2^; (4) physically active (>150 min of physical activity/week), no orthopedic limitations, non-smoker; and (5) low alcohol consumers (≤1 drink/day). Exclusion criteria were: (1) chronic diseases such as cardiovascular disease, diabetes and cancer, (2) currently following a fast food diet; (3) gastrointestinal problems such as bloating, nausea, indigestion, constipation and diarrhea. All participants lived in the Montreal region and spoke French and that the majority (87%) were University students with a low personal annual income (<$20,000) and were not married. The study was conducted in accordance with the Declaration of Helsinki and all procedures were approved by the Ethics Committee of the Université du Québec à Montréal (Trial registration: A-140063). All participants were fully informed about the nature, goal, procedures and risks of the study, and gave their informed consent in writing.

**Procedure:** A phone interview was conducted to screen for the aforementioned inclusion/exclusion criteria. After screening, each participant was invited to the Department of Exercise Science at the Université du Québec à Montréal in the fasting state at 8:00 a.m. for a series of tests. Upon their arrival, blood sampling, anthropometric, body composition, blood pressure and estimated maximal oxygen consumption measurements were performed. The exercise and dietary intervention began 48 h after metabolic testing. Furthermore, during the intervention a SenseWear armband was placed on the participant for the measurement of energy expenditure. Post testing was performed after 48 h of the last training session.

**Blood samples:** After an overnight fast (12 h), venous blood samples were collected and analyzed on the day of collection. Analyses were performed on the COBAS INTEGRA 400 analyzer (Roche Diagnostics, Laval, QC, Canada) for total cholesterol, HDL-cholesterol, triglycerides, glucose, aspartate aminotransferase (AST), alanine aminotransferase (ALT) and gamma-glutamyltransferase (GGT). LDL-cholesterol was calculated from the Friedewald equation [14]. Insulin levels were quantified in duplicate using a human insulin radioimmunoassay (Linco Research, Inc., St-Charles, MO, USA). Homeostasis model assessment (HOMA) = fasting insulin x fasting glucose/22.5 was then calculated [15]. Glycated hemoglobin (HbA1c) levels were measured by HPLC using the D-100 analyzer (Bio-Rad, Montreal, QC, Canada). Serum lipoprotein(a) (Lp(a)), apolipoprotein B (apoB) and high sensitivity (hs) C-reactive protein (hsCRP) were assessed by immunonephelometry on IMMAGE analyzer (Beckman-Coulter, Villepinte, France).

**Body composition:** Total body weight, fat mass, body fat percentage and lean body mass were measured using dual energy X-ray absorptiometry (General Electric Lunar Prodigy; standard mode; software version 12.30.008, Madison, WI, USA). Calibration was executed daily with a standard phantom prior to each test. In addition, standing height (±0.1 cm) was measured using a wall stadiometer (Perspective Enterprises, Michigan, USA). BMI = Body weight (kg)/Height (m^2^) was then calculated. Waist circumference was measured to the nearest 0.5 cm by using a non-elastic plastic tape with the participant standing upright.

**Blood pressure:** Systolic and diastolic sitting blood pressure was determined by an automatic sphygmomanometer machine (Spot Vital Signs^®^ Devices, Welch Allyn, Mississauga, ON, Canada). An appropriate cuff size was selected for each participant based on arm circumference. Conditions were carefully standardized: no talking, cuff on the left arm and 5 min of rest. Two measurements with 3 min of rest between measures were taken. The average of the two measures was reported.

**Estimated maximal oxygen consumption (VO_2_ max):** The Bruce Protocol on the treadmill was used to evaluate maximal oxygen consumption (VO_2_ max) [16]. The first stage began at a treadmill speed of 2.7 km/h and an incline of 10% gradient for 3 min. Thereafter, there was a progressive increase in the level of intensity (inclination and speed) every 3 min until voluntary exhaustion was reached. Maximal heart rate was recorded at exhaustion. The estimated VO_2_ max was calculated using the following validated prediction equation for men [17]: VO_2_ max (mL/kg/min) = 14.76 − (1.379 × T) + (0.451 × T2) − (0.012 × T3), where T = maximum time on the treadmill in minutes.

**Dietary intervention:** For 14 consecutive days, participants were subjected to an exclusive fast food diet from a popular fast food restaurant chain located in the Montreal area. Volunteers had to consume an extra value meal of their choice for breakfast, lunch and dinner. All meals for lunch and dinner consisted of a sandwich (e.g., Big Mac^®^, McChicken^®^, or Quarter Pounder with Cheese^®^), medium fries and a non-diet medium soft drink. As for breakfast, it consisted of a sandwich (e.g., Egg McMuffin^®^, or Sausage McMuffin^®^), hash browns and a small fruit drink or coffee. In addition, participants had the option to consume a muffin of their choice as a snack depending on their appetite. Each subject received a gift card to purchase all meals. Participants had to provide all receipts of the meals they had purchased to the researchers on a daily basis. This allowed determining exactly all of the foods each subject ingested. Dietary analyses were then conducted using the popular restaurant chain Nutrition Center’s website to determine total daily intake of protein, carbohydrate, fat, saturated fat, trans fat and sodium levels as well as total energy intake. In addition, all gastrointestinal problems such as bloating, nausea, indigestion, constipation and diarrhea were documented daily during the 14-day intervention. Each participant was instructed not to consume any other foods during the study. It should also be noted that a 2-week dietary intervention or less has been shown to be sufficient in inducing negative changes to the metabolic profile [10,18,19,20,21].

**Exercise intervention:** The high intensity interval training (HIIT) program was performed daily for 14 consecutive days on the treadmill. Each exercise session consisted of a 5 min warm-up, followed by 15 × 60 sec sprint intervals (~90% of maximal heart rate) interchanged with 60 sec active recovery (walking) and ended with 5 min cool-down. All exercise sessions were supervised by a kinesiologist and were performed at the Department of Exercise Science at the Université du Québec à Montréal. The HIIT program was adapted based on a previous study [22]. It should be noted that each participant was instructed not to perform any sports or other strenuous exercises during the study.

**Energy expenditure:** Total and physical activity energy expenditure as well as energy expenditure during HIIT were evaluated for 14 consecutive days using the portable mini SenseWear armband (Bodymedia, Pittsburgh, PA, USA). The portable armband uses a 3-axis accelerometer, a heat flux sensor, a galvanic skin response sensor, a skin temperature sensor, and a near-body ambient temperature sensor to capture data. These data as well as body weight, height, handedness and smoking status (smoker or non-smoker) are used to calculate energy expenditure. The armband was placed on the upper left arm (on the triceps at the mid-humerus point) of each volunteer. All participants were instructed to remove the armband only for bathing purposes or any water activity. The net output is a measure of energy expenditure (kilocalories) utilized by the participant across time. Data were extracted using the SenseWear professional software 8.1 (Bodymedia, Pittsburgh, PA, USA). This method of energy expenditure measurement has been validated by several studies [23,24,25].

**Statistical analysis:** The sample size calculation was based on the HOMA index considering potential changes of 0.3 ± 0.3 [10]. At the end of the study, fourteen participants were needed for a power of 80% and an alpha error of 0.05. Because we predicted a 15% dropout rate, 16 subjects were recruited at the start of the study. Results are presented as means ± SD. We first verified the normality of the distribution of variables with the Skewness test and found that all variables were normally distributed. To identify differences before and after the intervention, we conducted a repeated measures analysis of variance (ANOVA) with a within-subjects factor of time. The Bonferronni correction was applied. We also calculated the effect size (mean difference pre-post intervention/SD) for all variables. Statistical analysis was performed using IBM SPSS Statistics for Windows, Version 22 (IBM Corp., Armonk, NY, USA). Statistical significance was set at *p* ≤ 0.05.

## 3. Results

Sixteen participants were recruited in the study. However, one subject dropout due to knee discomforts. Therefore, a total of 15 participants completed the study and were included in the analysis. Mean attendance for the exercise sessions was 100% and mean adherence to the fast food diet was 99% in the participants that completed the study. Furthermore, 13 subjects did not report any gastrointestinal incidents during the 14-day intervention, whereas one participant reported three occurrences and one other reported one occurence. All of the gastrointestinal episodes were minor.

The fast food diet and energy expenditure profiles of the participants during the intervention are presented in Table 1. Briefly, mean energy intake and energy expenditure of the participants was 3441 ± 337 kcal/day and 3503 ± 373 kcal/day, respectively.

Table 2 shows the physical characteristics of the participants before and after the intervention. We found significant improvements for body fat percentage (effect size (ES): 0.70), fat mass (ES: 0.54), lean body mass (ES: 0.59) and estimated VO_2_ max (ES: 0.67) after the intervention (*p* ≤ 0.05). No significant differences were observed for total body weight, BMI, and waist circumference.

Cardiometabolic characteristics of the participants before and after the intervention are presented in Table 3. We noted significant improvements for fasting glucose (ES: 0.81), Lp(a) (ES: 0.63) and hsCRP (ES: 0.59) after the intervention (*p* ≤ 0.05). However, HDL-cholesterol significantly decreased after the intervention (ES: 0.96) (*p* < 0.01). We also found a lower tendency for total cholesterol (*p* = 0.06) and AST levels (*p* = 0.08). No changes were observed for all the other cardiometabolic variables.

## 4. Discussion

In the documentary “Super Size Me”, the main character of the film gains 11 kg of body weight and deteriorates his health after eating fast food from a popular fast food restaurant chain three times a day for 30 days and this without exercising. Results of the present showed that a similar fast food diet for 14 consecutive days in combination with a HIIT program did not, in large part, deteriorate the cardiometabolic profile. In fact, we noted improvements in body composition, estimated VO_2_ max, fasting glucose, Lp(a) and hsCRP levels. The magnitudes of these improvements were medium (effect sizes: 0.54–0.70), except for fasting glucose, which was large (effect size: 0.81). However, HDL-cholesterol significantly decreased (large effect size: 0.96) after the intervention. The mechanism that could explain this phenomenon is unclear. Indeed, there is evidence to suggest that the triglycerides/HDL-cholesterol ratio may be a better clinical tool to identify cardiometabolic risk factors such as insulin sensitivity instead of triglycerides or HDL-cholesterol alone [26,27,28]. In the present study, the triglycerides/HDL-cholesterol ratio did not change after the intervention. In addition, a recent study has questioned if HDL-cholesterol may be an independent cardiovascular risk factor [29]. Furthermore, no significant changes were observed for all the other cardiometabolic parameters such as blood pressure, hepatic enzymes, apoB and insulin resistance. It should be noted that the primary outcome (HOMA index) in our study was not altered after the intervention. Only certain secondary outcomes were significant and should, therefore, be considered as exploratory. As such, future studies are needed to further test these secondary outcomes. Collectively, results of the present suggest that HIIT may be a promising strategy to protect against weight gain and cardiometabolic abnormalities when consuming fast foods.

Similar results have been reported in animal studies who also observed the protective effects of exercise training against an unhealthy dietary regimen [30,31,32]. For example, eight weeks of a high fat-diet (40% kcal) in combination with exercise training on the treadmill (five times/week) completely prevented the accumulation of fat in the liver of rats [30]. The same group, in another study [31], showed that introducing exercise training during the course of a high fat diet significant decreased visceral and subcutaneous fat content, plasma free fatty acids and leptin levels. Moreover, Pimenta et al. [32] noted that eight weeks of HIIT (swimming) minimized the detrimental effects of a high fat diet on cardiometabolic risk factors in ovariectomized mice. Finally, a recent study reported that a single bout of aerobic exercise protected against the inflammatory response of a high fat meal in young men [21].

This study has several limitations. First, our small sample size was only composed of healthy physically active young men. Therefore, our findings are limited to this population. Second, there was no control group due to ethical reasons as well as there is evidence to suggest that a fast food diet is associated with negative health outcomes such as weight gain, increases in waist circumference, insulin resistance, triglycerides and a decrease in HDL-cholesterol [5,6,7,8,33]. It should also be noted that the participants did not want to be in a control group (without exercise) since their goal was not to deteriorate their health. Third, the duration of the intervention was short-termed. Whether HIIT is efficient in protecting the general population (e.g., women, less active individuals, and elderly) from a fast food diet, should be examined with a larger sample size in a randomized controlled study with longer interventions. Indeed, a potential hypothesis would be that HIIT could counteract the detrimental effects of a fast food diet on the cardiometabolic profile in long-term studies due to its strong effect in improving insulin sensitivity, blood pressure and the lipid profile [11,12,13].

## 5. Conclusions

In summary, HIIT seems to protect, in large part, the cardiometabolic profile against the potential negative effects of a fast food diet in young healthy men. Therefore, these findings contradict the popular believe of the general population. Indeed, our results may be useful for clinical and practical purposes. It is important to educate health care professionals and athletes/coaches regarding the potential protective effects of HIIT against a fast food diet. Nevertheless, these findings should be considered preliminary, but they may stimulate interest for additional research on the impact of a fast food diet in combination with HIIT on metabolic diseases in different populations.

## Figures and Tables

**Table 1:** Fast food diet and energy expenditure profiles of the participants (*n* = 15) during the intervention.

Variables	Mean ± SD
Total energy intake (kcal/day)	3441 ± 337
Protein (g/day)	100.5 ± 9.5
Protein (%)	11.7 ± 0.7
Carbohydrate (g/day)	421.8 ± 46.5
Carbohydrate (%)	49.1 ± 2.2
Fat (g/day)	150.3 ± 15.6
Fat (%)	39.3 ± 1.7
Saturated fat (g/day)	40.8 ± 4.6
Trans fat (g/day)	2.6 ± 0.4
Sodium (mg/day)	4724 ± 405
Total energy expenditure (kcal/day)	3504 ± 373
Physical activity energy expenditure (kcal/day)	1588 ± 371
Energy expenditure during HIIT (kcal/day)	413.5 ± 29.8

Values are mean ± SD; HIIT: high intensity interval training.

**Table 2:** Physical characteristics of the participants (*n* = 15) before and after the intervention.

Variables	Pre	Post	P value	Effect size
Age (years)	23.8 ± 2.8	-	-	-
Height (m)	1.79 ± 4.8	-	-	-
Total body weight (kg)	75.8 ± 7.7	75.9 ± 7.7	0.61	0.13
Body mass index (kg/m^2^)	23.4 ± 2.2	23.4 ± 2.2	0.33	0.25
Waist circumference (cm)	81.4 ± 5.7	81.7 ± 5.1	0.52	0.18
Body fat (%)	13.5 ± 3.5	13.1 ± 3.6	0.02	0.70
Fat mass (kg)	10.4 ± 3.6	10.2 ± 3.6	0.05	0.54
Lean body mass (kg)	62.0 ± 4.9	62.4 ± 5.0	0.03	0.59
Estimated VO_2_ max (mL/kg/min)	55.8 ± 8.3	57.5 ± 7.1	0.03	0.67

Values are mean ± SD.

**Table 3:** Cardiometabolic characteristics of the participants (*n* = 15) before and after the intervention.

Variables	Pre	Post	*p* Value	Effect Size
Systolic blood pressure (mmHg)	116.7 ± 9.7	115.5 ± 9.4	0.62	0.13
Diastolic blood pressure (mmHg)	68.6 ± 4.7	70.8 ± 4.7	0.10	0.44
HbA1c (%)	5.0 ± 0.3	5.0 ± 0.2	0.19	0.34
Fasting glucose (mmol/L)	4.63 ± 0.3	4.39 ± 0.3	0.007	0.81
Fasting insulin (pmol/L)	53.3 ± 34	46.6 ± 22	0.45	0.22
HOMA index	1.83 ± 1.2	1.54 ± 0.7	0.35	0.27
Total cholesterol (mmol/L)	4.10 ± 0.62	3.83 ± 0.64	0.06	0.52
LDL-cholesterol (mmol/L)	2.26 ± 0.58	2.10 ± 0.54	0.13	0.41
HDL-cholesterol (mmol/L)	1.48 ± 0.2	1.35 ± 0.2	0.002	0.96
Triglycerides (mmol/L)	0.80 ± 0.3	0.84 ± 0.2	0.67	0.11
Triglycerides/HDL-cholesterol	0.56 ± 0.3	0.63 ± 0.2	0.22	0.33
ApoB (g/L)	0.69 ± 0.1	0.64 ± 0.1	0.11	0.46
Lp(a) (g/L)	0.14 ± 0.1	0.12 ± 0.1	0.04	0.63
hsC-reactive protein (mg/L)	1.13 ± 1.4	0.32 ± 0.1	0.047	0.59
Alanine aminotransferase (U/L)	23.3 ± 14.5	20.7 ± 3.3	0.50	0.18
Aspartate aminotransferase (U/L)	32.5 ± 15.6	24.6 ± 5.0	0.08	0.5
γ-glutamyltransferase (U/L)	16.8 ± 6.5	15.9 ± 5.1	0.32	0.28

Values are mean ± SD.
